# Gut microbiota composition in the sympatric and diet‐sharing *Drosophila simulans* and *Dicranocephalus wallichii bowringi* shaped largely by community assembly processes rather than regional species pool

**DOI:** 10.1002/imt2.57

**Published:** 2022-10-13

**Authors:** Yu‐Xi Zhu, Run Yang, Xin‐Yu Wang, Tao Wen, Ming‐Hui Gong, Yuan Shen, Jue‐Ye Xu, Dian‐Shu Zhao, Yu‐Zhou Du

**Affiliations:** ^1^ Department of Entomology, College of Plant Protection Yangzhou University Yangzhou China; ^2^ The Key Laboratory of Plant Immunity, Jiangsu Provincial Key Lab for Organic Solid Waste Utilization, Jiangsu Collaborative Innovation Center for Solid Organic Wastes, Educational Ministry Engineering Center of Resource‐saving fertilizers Nanjing Agricultural University Nanjing China; ^3^ Bureau of Agriculture and Rural Affairs of Binhu District of Wuxi Wuxi China; ^4^ Entomology and Nematology Department University of Florida Gainesville Florida USA

**Keywords:** community assembly, *Dicranocephalus wallichii bowringi*, *Drosophila simulans*, gut microbiota, microbial source‐tracking

## Abstract

Clarifying the mechanisms underlying microbial community assembly from regional microbial pools is a central issue of microbial ecology, but remains largely unexplored. Here, we investigated the gut bacterial and fungal microbiome assembly processes and potential sources in *Drosophila simulans* and *Dicranocephalus wallichii bowringi*, two wild, sympatric insect species that share a common diet of waxberry. While some convergence was observed, the diversity, composition, and network structure of the gut microbiota significantly differed between these two host species. Null model analyses revealed that stochastic processes (e.g., drift, dispersal limitation) play a principal role in determining gut microbiota from both hosts. However, the strength of each ecological process varied with the host species. Furthermore, the source‐tracking analysis showed that only a minority of gut microbiota within *D. simulans* and *D. wallichii bowringi* are drawn from a regional microbial pool from waxberries, leaves, or soil. Results from function prediction implied that host species‐specific gut microbiota might arise partly through host functional requirement and specific selection across host–microbiota coevolution. In conclusion, our findings uncover the importance of community assembly processes over regional microbial pools in shaping sympatric insect gut microbiome structure and function.

## Highlights

- The diversity, composition, and network of gut microbiota differed between the sympatric and diet‐sharing *Drosophila simulans* and *Dicranocephalus wallichii bowringi*.
- Host species shape the bacterial and fungal community in two insect hosts by altering the relative contribution of community assembly processes.
- A minority of gut microbiota within *D. simulans* and *D. wallichii bowringi* are drawn from a regional microbial pool from waxberries, leaves, or soil.
- The composition of insect gut microbiota is driven by community assembly processes in a host species‐dependent manner more than regional microbial pools.

## Graphical abstract

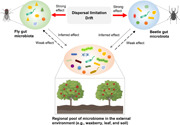

## INTRODUCTION

Animal guts harbor a particularly dense and diverse community of microbes [1, 2]. These microbes are closely tied to the physiology, ecology, and evolution of their hosts [3, 4], frequently performing key services such as maintaining fitness [5], digestion [6], detoxification [7, 8], immunity [9, 10], compensating nutrients [11], and stress responses [12]. Despite this being critical for the vast majority of animals [2, 9, 13], the functions and forces that sculpt gut microbial communities in insects remain largely unexplored. Deciphering the processes shaping gut microbiota composition is a premise for designing microbial strategies to control insect pests or protect the health of economically favorable insects.

Regional species pools are well known as a mediator of animal microbiomes [14–16]. Similar microbiome compositions may be traced to similar diets or shared environments; many insects directly acquire microbes from their diets or surroundings [17–19]. For example, bees acquire microbiota from their floral environments via foraging [20]. Diet can also rapidly and reproducibly alter the gut microbiome in many insects [21]. Species such as foliar‐feeding caterpillars lack resident gut microbes and obtain microbiomes from the soil instead of their dietary plants [19, 22]. Synthesizing these results, it can be seen that gut microbes can be derived from adjacent trophic levels or any component of the trophic network.

In parallel, neutral theory highlights the critical role of microbial community assembly processes, particularly stochastic processes, in driving the microbiomes to converge or diverge [23–25]. More generally, deterministic and stochastic processes work in conjunction in microbiome assembly, but the relative contribution of these ecological processes (e.g., diversification, selection, dispersal, and drift) in shaping host microbiomes is likely to vary among systems [26–28]. For example, recent work on honeybee gut microbiota has shown that stochastic processes like drift were the critical factor [28]. In contrast, other studies point to a more substantial effect of deterministic processes like selection [29]. Furthermore, the magnitude of these ecological processes is also closely related to the host species, diet, or environment [26, 27, 30, 31].

A central open question, therefore, is whether the regional microbial pool or community assembly processes dominate in shaping the microbiota. The resolution of this question has been hampered by the complex and multilayered interactions between myriad factors in a natural setting. To address this challenge, we explored the gut bacterial and fungal communities in two different insect species, the fruit fly *Drosophila simulans* and the flower beetle *Dicranocephalus wallichii bowringi*, which share a common diet and habitat. Adult *D. simulans* and *D. wallichii bowringi* feed on ripe fruits including waxberry using sponging and chewing mouthparts, respectively. Although the microbiota of both *Drosophila* [29, 32, 33] and of beetles [34–36] are well studied, there are less data on the sources and assembly of gut microbiota in wild populations of these insects. This study addresses this gap by testing whether these insect gut microbiomes were derived from regional microbial pools or community assembly processes. We first investigate the similarity between microbiomes from different types of samples and assess whether the composition of gut microbiota of these insects could be attributed to those from their diet (i.e., waxberry) or environment (leaf or soil). Second, we evaluate the assembly mechanisms of the microbiomes in the two insect species. Finally, we discuss the potential functions provided by the gut microbiome to the two insect species.

## RESULTS

### Microbial diversity, composition, and networks among host types

**Figure 1:**
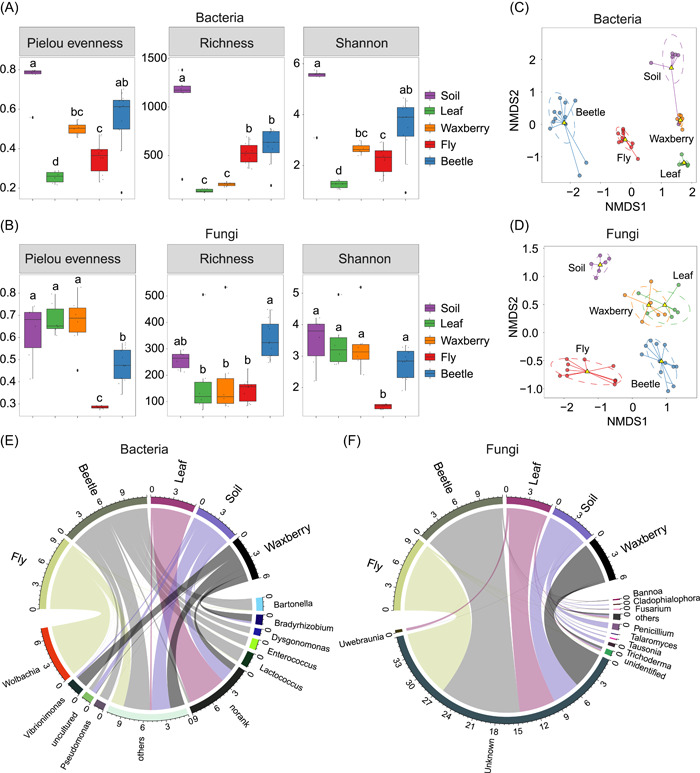
Diversity and structure of microbial communities in *D. simulans*, *D. wallichii bowringi*, waxberry, leaves, and soil. Box and whisker plots of three α‐diversity indices (Pielou evenness, Richness, and Shannon diversity index) of bacterial (A) and fungal (B) communities in each group. Different letters above the whiskers denote significant differences between each group determined with analysis of variance tests (*p* < 0.05). Nonmetric multidimensional scaling (NMDS) of bacterial (C) and fungal (D) communities, with clustering based on Bray–Curtis similarities. Relative abundances of bacterial genera (E) and fungal genera (F) in microbial composition among *D. simulans*, *D. wallichii bowringi*, waxberry, leaves, and soil.

The Shannon diversity indices of bacterial and fungal communities significantly differed in soil, leaves, waxberries, flies, and beetles (Figure 1A). Remarkably, in the two insect species, host species (*F* = 11.25, *p* = 0.004) but not host sex (*F* = 3.13, *p* = 0.09) exhibited significant impact on gut bacterial community diversity, while gut fungal diversity was strongly influenced by both host species (*F* = 114.90, *p* < 0.0001) and host sex (*F* = 6.66, *p* = 0.02) (Supporting Information: Table S1). Nevertheless, the fly had a significantly lower diversity of both bacterial and fungal microbes in its gut compared to the beetle (bacteria: *p* < 0.01; fungi: *p* < 0.0001) (Figure 1A).

Both bacterial and fungal communities varied in composition strongly based on the type of sample (soil, leaf, waxberry, fly gut, and beetle gut) (PERMANOVA, bacteria: multi‐response permutation procedures (MRPP) = 0.40, *p* < 0.001; fungi: MRPP = 0.55, *p* < 0.001; Figure 1C,D). The fly gut was occupied primarily by the bacterial phylum Proteobacteria, whereas the beetle gut was dominated by Firmicutes (Supporting Information: Figure S1A). In terms of fungi, both fly and beetle guts consisted of almost entirely unclassified groups (Supporting Information: Figure S1B). At the genus levels, the bacteria *Wolbachia*, *Acetobacter*, *Commensalibacter*, and the fungi *Fusarium*, *Naganishia*, and *Penicillium* were more abundant in the fly guts. In contrast, the bacteria *Lactococcus*, *Weissella*, *Bartonella*, *Pseudomonas*, *Enterococcus*, and the fungi *Bannoa*, *Kwoniella*, and *Hasegawazyma* were more abundant in the beetle guts (*p* < 0.05 for all cases; Figure 1E,F, and Supporting Information: Figure S2).

Network analysis revealed that the complexity of bacterial and fungal co‐occurrence networks was significantly higher in leaves (Average degree: bacterial: 32.90; fungal: 144.55) and waxberries (bacterial: 26.73; fungal: 105.44) than in the fly (bacterial: 5.39; fungal: 93.20) and beetle guts (bacterial: 7.06; fungal: 7.27) (Supporting Information: Figure S3 and Table S2). Similarly, the number of connections in both bacterial and fungal communities was higher in leaves (bacteria: 6811; fungi: 35,197) and waxberries (bacteria: 5332; fungi: 25,781) than in flies (bacteria: 833; fungi: 22,367) or beetles (bacteria: 1568; fungi: 1531) (Supporting Information: Figure S3 and Table S2). The gut bacterial network in the beetle was more complex than that in the fly, but the fungal networks were the opposite.

### The networks between gut bacteria‐fungi in each insect species

**Figure 2:**
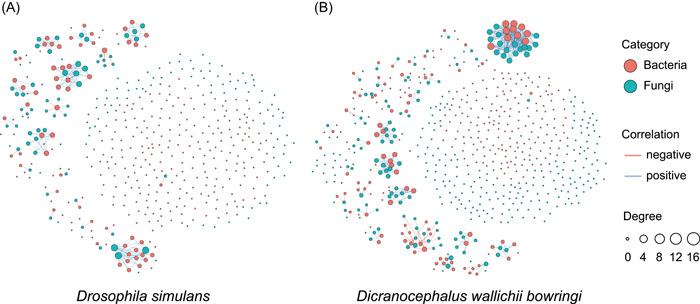
Co‐occurrence networks of gut bacteria and fungi in *D. wallichii bowringi* (A) and *D. simulans* (B). Edges represent statistically significant Spearman correlations (*ρ* > |0.6|, *p* < 0.05), whereas blue and red lines, respectively, indicate significant positive and negative correlations. Relative abundances of OTUs in each microbial community are represented by the sizes of the points.

We tested for interactions between gut bacteria and fungi in both insect species. There was no significant correlation of α‐diversity between bacteria and fungi in *D. wallichii bowringi* (Shannon: *r* = −0.51, *p* = 0.13; Richness: *r* = −0.47, *p* = 0.17) nor *D. simulans* (Shannon: *r* = −0.33, *p* = 0.30; Richness: *r* = −0.48, *p* = 0.12) (Supporting Information: Figure S4), suggesting that bacteria and fungi may have different ecological niches in the host. However, network analyses showed that certain bacterial symbionts were significantly correlated with fungi species in the same host insect (Figure 2 and Supporting Information: Table S3). The number of connections between gut bacteria and fungi was higher in the beetle (366) than in the fly (185). As well, the average degree of the network of bacteria and fungi was higher in beetle than in the fly (3.83 vs. 3.25) (Figure 2 and Supporting Information: Table S4). These results imply that some microbes may have mutualistic interactions with fungi in either the fly or the beetle.

### Shared microbes among soil, leaves, fruit, and two insects

In total, we recovered 4787 bacterial OTUs and 3394 fungal OTUs from all samples. The soil contained the most bacterial OTUs (*n* = 2258), followed by the gut of the beetle (*n* = 1827) and the fly (*n* = 1766) (Supporting Information: Figure S5). In contrast, the beetle gut contained the most fungal OTUs, followed by the fly gut (Supporting Information: Figure S5). A total of 105 bacterial and 25 fungal OTUs were shared between the gut of beetle and fly (Supporting Information: Figure S5).

**Figure 3:**
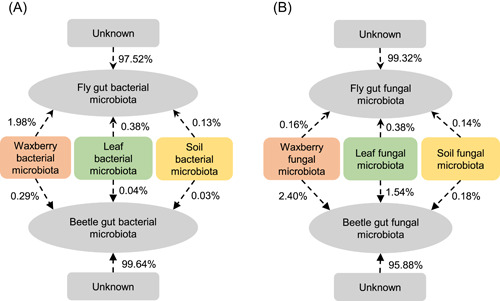
Potential sources of gut bacteria (A) and fungi (B) in the two wild, sympatric insect species. Numbers next to arrows indicate the proportion of the fly *D. simulans* and beetle *D. wallichii bowringi* microbes potentially derived primarily from the microbiota of their diet waxberry or surrounding leaves and soil.

Microbial source tracking revealed that the fly and beetle species studied here respectively acquired only 1.98% and 0.38% of gut bacteria, and only 0.16% and 2.40% of gut fungi from the waxberry bacterial pool (Figure 3). Remarkably, in both *D. simulans* and *D. wallichii bowringi*, the large majority of the gut microbiomes of both bacteria and fungi were not derived from any of their diet (waxberry) or surrounding environmental microbiota (leaves or soil) (fly: bacteria: 97.52%, fungi: 99.32%; beetle: bacteria: 99.64%, fungi: 95.88%; Figure 3).

### Gut bacterial and fungal community assembly of the subject fly and beetle species

**Figure 4:**
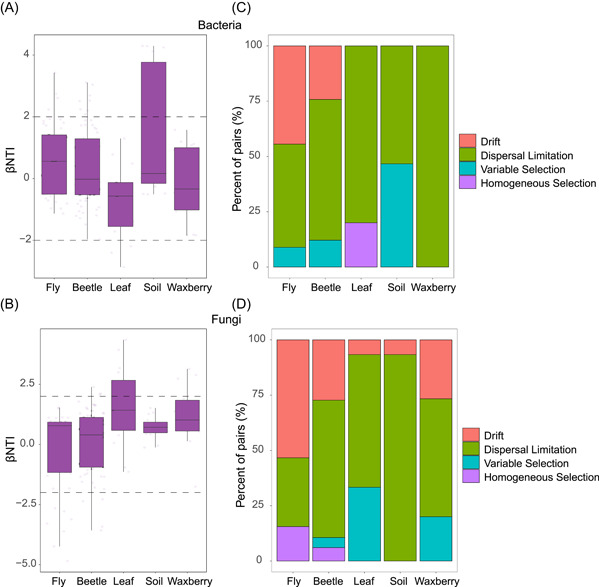
Mechanisms of microbial community assembly in *D. wallichii bowringi*, *D. simulans*, waxberry, leaves, and soil. Box and whisker plots of contributions of deterministic (|βNTI| ≥ 2) and stochastic processes (|βNTI| < 2) on bacterial (A) and fungal (B) community assembly in each group. The relative contributions of ecological processes in driving the bacterial (C) and fungal (D) assembly in each group.

The relative contribution of different ecological processes in shaping microbiota assembly in the two insects were quantified with null model analyses. Results showed that bacterial and fungal community assembly in each of the host types was primarily driven by stochastic processes (−2 < βNTI < 2) (Figure 4A). In both *D. simulans* and *D. wallichii bowringi*, dispersal and drift were the top factors driving both bacterial and fungal communities, followed by variable selection for bacteria and homogeneous selection for fungi (Figure 4B). However, drift had a higher relative contribution to the assembly of both bacterial and fungal communities in the gut of flies (bacteria: 44.44%; fungi: 53.33%) than in the beetles (bacteria: 24.24%; fungi: 27.27%). On the other hand, the relative influence of dispersal limitation was higher for communities in beetles (bacteria: 63.64%; fungi: 62.12%) than those in flies (bacteria: 46.67%; fungi: 31.11%) (Figure 4B). Collectively, these results demonstrate that stochastic processes, including drift and dispersal, dominantly drive the community assembly of the bacteria and fungi, and that their relative influence is dependent on the taxonomy of the host.

### Functional inference of gut microbiota in the fly and beetle

**Figure 5:**
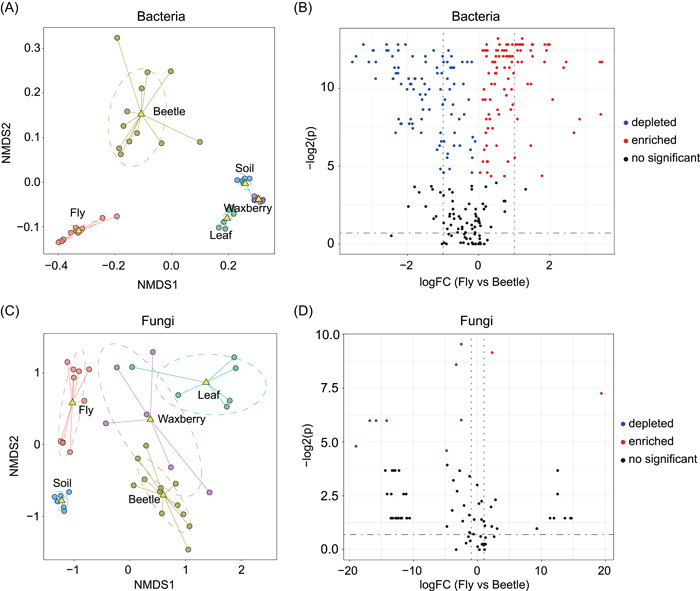
Functional predictions of bacteria and fungi varied among different sample types. Nonmetric multidimensional scaling ordination (NMDS) showing that potential functions of the bacteria (A) and fungi (C) clustered by sample type using Bray–Curtis dissimilarity distance. Boxplots representing significantly different potential functions of the bacteria (B) and fungi (D) between *D. simulans* and *D. wallichii bowringi*. The negative log2 of adjusted *p* values plotted against the logFC between *D. simulans* and *D. wallichii bowringi*. Red, blue and gray dots indicate the enriched, depleted, and nonsignificant functional categories, respectively. Detailed information for these functional categories are in the Supporting Information: Table S5.

The bacteria identified were rich in genes associated with metabolic pathways, microbial metabolism in diverse environments, carbon metabolism, fatty acid metabolism, peptidoglycan biosynthesis, the citrate cycle, pentose phosphorylation pathways, and the two‐component system (Supporting Information: Figure S6A). Functions of the abundant bacterial genes varied strongly between the fly gut and the beetle gut (Pair Adonis, MRPP = 0.071, *p* < 0.001) (Figure 5A). Specifically, there were significant differences between the fly and beetle guts in terms of the main function of genes involved in ABC transporters, phosphotransferase systems, amino acid metabolism, and purine metabolism (Figure 5B and Supporting Information: Table S5).

Based on predictions using FUNGuild analysis, the major functions of the fungal communities were classified as Pathotroph, Saprotroph, and Symbiotroph (Supporting Information: Figure S6B). There were significant differences between the fly and beetle guts in terms of the main function of abundant fungal genes involved in Saprotrophy, plant pathogens, fungal parasites, and animal pathogens (Pair Adonis, MRPP = 0.499, *p* < 0.001) (Figure 5C,D and Supporting Information: Table S5). These results suggest that gut microbiota may provide hosts with diverse and specific functions.

## DISCUSSION

Theoretically, gut microbiota can be thought of as a local community colonized from a regional microbiome pool [15, 17, 52]. However, microbes colonizing a host gut are often limited by a unique set of microbial community assembly processes [23, 32]. The relative importance of these underlying forces, including the microbiome pool and community assembly processes, in shaping insect gut microbiota composition remains largely controversial. We found in this study that the diversity, composition, and network properties of gut bacterial and fungal communities differed between two insect species living in sympatry and sharing a common diet, suggesting that host species can strongly affect the gut microbiota. Interestingly, variation in the microbial communities between two insect species is likely driven by variation in relative influences of community assembly processes and is independent of the regional environmental microbial pool (i.e., diets, surrounding plants, and soil).

**Figure 6:**
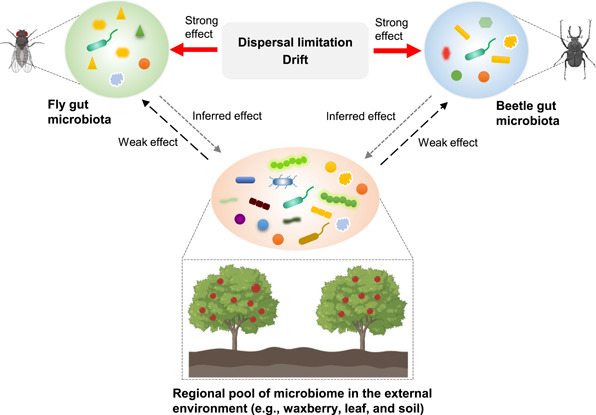
An illustration of the sources and assembly of microbiota associated with the gut of *D. simulans* and *D. wallichii bowringi* and the regional pool of microbiomes in the external environment (e.g., waxberry, leaf, and soil) that they may draw from. Shapes represent different types of microbes. Different arrows indicate the extent of each factor in shaping the microbial community: solid arrows indicate a strong effect, while two types of dotted arrows show a weak effect or inferred effect.

We found that only a tiny minority of gut bacterial and fungal microbiota within *D. simulans* and *D. wallichii bowringi* might draw from a regional microbial pool from the environment (waxberry, leaf, and soil), indicating that the regional pool plays only a weak role in shaping the gut microbiome of these species (Figure 6). This suggests that pest control methods that attempt to disrupt the balance of insect gut microbiomes by changing regional microbial pools may only have limited efficacy [53, 54]. Furthermore, a recent publication suggests that aboveground insects reshape leaf microbiota [55]. This raises an interesting question of whether these insects also change the regional microbial pool. The complex interactions that could exist among multiple niche networks require further investigation.

Previous studies in wild flies and beetles have shown that gut bacterial and fungal communities are strongly linked to host diet and habitat [32, 35], which implies that the regional pool, in particular diet, is a primary mechanism of microbiota acquisition for many insects [56, 57]. Controlled experiments using laboratory caterpillars also suggested that foliar‐feeding insects may directly acquire microbe diversity from their diets or the surrounding environment (e.g., soil) via a hitchhiking effect through the network [19]. Our findings in two species, one Diptera and one Coleoptera, appear to directly contrast these results, showing that despite sharing a diet, the two species displayed large differences in both bacterial and fungal community compositions, and that gut microbiotas were host‐species specific. On a broader scale, our results do agree with the observation that host species, more so than environmental factors (e.g., diet), shape gut microbial diversity and composition [33, 58, 59, 60, 61, 62]. Therefore, we speculate that a common diet may not be enough to disturb species‐specific differences in the gut microbiota. However, we cannot completely rule out the possibility that these insects also fed on other fruits before transferring to waxberry in their natural environment.

Multiple mechanisms could explain our findings of host species being dominant in driving gut microbiota. First, the insect gut is a major filter with unique morphological and physiological conditions, and many microbes from the environmental pool may fail to colonize a host gut [15, 63]. Variations in size, morphology, and physiology of the gut between these two insect species might lead to differences in microbial composition. Second, coexisting microbes may compete for limited resources and space [64]. Variation in the outcomes of microbe‐microbe competition could contribute to gut microbiota variation among insect groups [65, 66]. In our study, bacteria–fungi interactions in the same host gut were also observed. In particular, heritable endosymbionts (e.g., *Wolbachia*) that have been found to contribute to gut community structure in *D. melanogaster* [67] were highly abundant in *D. simulans* studied here. Thus, we speculate that interaction between microbes is an essential factor that alters community diversity and composition. Nonetheless, further investigation is required to explore these possibilities. Finally, from the ecological perspective, neutral processes, which are independent of host species and traits, and microbial assembly processes, which often vary between host species, cause variation in microbiome composition between different insect species [23].

We found stochastic processes like drift and dispersal limitation to be the primary influence on bacterial community assembly within the gut of both *D. simulans* and *D. wallichii bowringi* (Figure 6). Similar results demonstrating the importance of stochastic forces were also observed in studies on gut microbial communities in zebrafish [68]. On the other hand, several studies on gut microbiota assembly in other insect species have pointed to a deterministic process, likely selection, playing a larger role in shaping community structure [28–30]. These contrasting observations indicate that the relative contribution of each ecological process varies strongly with host species. Recent studies also suggested that mechanisms of community assembly were related to other environmental factors such as geographic variables [28, 31]. In our study, the magnitude of effects of drift and dispersal limitation differed across host taxa sharing the same environment. Dispersal limitation causes communities to diverge, whereas drift disperses microbiota communities [23, 25]. This partially explains why gut microbiota networks and composition can differ substantially between the two species. A recent study on honeybee gut bacterial community assembly speculated that, from a long‐term coevolution perspective, deterministic processes tend to alter the direction of coevolution while stochastic processes are instead the driving forces progressing coevolution [28]. In most cases, coevolution tends to drive symbiotic relationships into mutualistic, win‐win relationships.

In most cases, despite taxonomical and functional diversity, gut microbiota serves a conserved set of symbiotic roles across various host animals, mainly supporting their metabolism, reproduction, pathogen resistance, and immune system functions [69, 70]. In a diverse range of insect taxa including Hymenoptera, Diptera, and Hemiptera, gut microbiota plays pivotal roles in the host's physiological functions such as plant polysaccharide digestion and pathogen defense [1, 6, 10, 70, 71]. Thus, understanding the functions of gut microbiota provides candidate molecular targets for disrupting these symbioses to control pests or protect the health of economic insects [1]. Given that both *D. simulans* and *D. wallichii bowringi* feed on waxberry for the supplement of sugar, amino acids, and other essential nutrients for their growth at specific stages of development [72], this partly explains why the majority of gut bacteria are involved in carbon and amino acid metabolism. This raises a possible question of whether host‐specific gut microbiota arises partly through host functional requirement and selection across host–microbiota coevolution. However, the functional relevance of bacteria–fungi interactions in the insect gut, in terms of their physiological consequences for the host, remains unknown. The extent of functional redundancy in these microbial communities and how this affects measures of diversity and niche overlap is also unclear [73].

Remarkably, the dominant microbe in the gut of *D. simulans* was *Wolbachia*, a common symbiont in insects and other arthropods and well known for manipulating host reproduction via various phenotypic effects [74, 75]. Previous studies have also shown that *Wolbachia* could affect host immunity [9, 10], influence host environmental adaptation [12, 76], alter host–plant interactions [77, 78], and influence other ecological and physical functions [3, 4]. Some strains of *Wolbachia* have also been suggested to mediate reproduction, fitness, and immune responses in *D. simulans* [79–82]. Knowing the potential of *Wolbachia* symbiosis as a tool for controlling pests [83], the interactions between *Wolbachia* and *D. simulans* demand further exploration within an environmental context.

## CONCLUSIONS

In summary, we find that between two sympatric insect species sharing a diet, gut microbiota remains highly species‐specific and that while stochastic processes played a primary role in driving microbial community assembly, the majority of gut microbiota is unlikely to be randomly acquired from a common microbial pool. Thus, we suggested that gut microbial diversity patterns and functions may be closely related to community assembly processes in a host species‐dependent manner, rather than drawing from regional microbial pools. We also discussed the potential functions of gut microbiota in both host species, and an important future goal will be to affirm specific functions of core gut bacteria and fungi in these two insects.

## METHODS

### Sample collection

All specimens were collected from a strawberry orchard in Wuxi in June 2021. We randomly selected a total of six sympatric strawberry trees. The adult horned flower beetle *D. wallichii bowringi* and adult fruit fly *D. simulans* were collected from ripe waxberries (Supporting Information: Figure S7). The corresponding waxberry tissues and surrounding leaves of these insect samples were also collected. Samples were preserved in 100% ethanol and then stored at −20°C. For soil sampling, we randomly selected four plots around the same plant. Soil (10–30 g FW for each plot) was collected as four subsamples, which were thoroughly mixed into one biological sample for each plant.

Individuals of the adult *D. simulans* and adult *D. wallichii bowringi* were surface sterilized with 100% ethanol, and then with distilled water three times. The entire gut was dissected from each individual under sterile conditions with flame‐sterilized forceps in 1× phosphate‐buffered saline (PBS) and washed three times with sterile water to egest the contents from their guts. Before DNA extraction, the gut of each insect was stored separately in sterile tubes with 40 μl H_2_O at −20°C.

### DNA extraction, internal transcribed spacer 1/2 (ITS1/2), and 16S **ribosomal ribonucleic acid** (rRNA) amplicon sequencing

DNA was extracted from individually dissected guts using a DNeasy blood and tissue kit (Qiagen) following protocol by the manufacturer. For DNA extraction from samples of the leaves, waxberry, and soil, the DNeasy Plant or PowerSoil Kit (Qiagen) was used accordingly. To evaluate the quantity and quality of the extracted DNA, electrophoresis with 1% agarose gel and an ultraviolet spectrophotometer (Nanodrop 2000) were used, respectively.

After DNA extraction, the community compositions of bacteria and fungi in samples of leaves, soil, waxberries, and the two insect species (*D. simulans* and *D. wallichii bowringi*) were analyzed using parallel 16S rRNA gene amplicon and internally transcribed space region high‐throughput sequencing [37, 38]. The primers 341F (5′‐CCTAYGGGRBGCASCAG‐3′) and 806R (5′‐GGACTACNNGGGTATCTAAT‐3′) were used to amplify the V3–V4 region of the bacterial 16S rRNA gene, whereas the fungal ITS region was amplified using primers ITS1/ITS2 (ITS1F: 5′‐CTTGGTCATTTAGAGGAAGTAA‐3′) and ITS2R (5′‐GCTGCGTTCTTCATCGATGC‐3′).

Polymerase chain reaction (PCR) amplifications were performed on ABI GeneAmp® 9700. The mixtures for the amplification process contained 4 μl of 5× FastPfu Buffer, 2 μl of dNTPs (2.5 mM), 0.8 μl of each primer (5 μM), 0.4 μl of TransStart Fastpfu DNA Polymerase, 10 ng of template DNA, and lastly ddH_2_O to reach a total volume of 20 μl. Three replicates for each sample were amplified using the following protocol: first, samples were heated for 5 min at 95°C, then they underwent 30 cycles of 30 s of denaturation (95°C), 30 s of annealing (55°C), and 45 s of extension (72°C); this was followed by a final 10‐min extension phase at 72°C. The resultant amplicons were extracted from 2% agarose gels and purified using the AxyPrep DNA Gel Extraction Kit (Axygen Biosciences) according to the manufacturer's instructions. Products for each sample were then pooled in equimolar concentrations before sequencing analysis. Truseq DNA PCR‐Free Library Preparation Kits were used to construct sequencing libraries for the analyses. Two libraries, one for bacterial and one for fungi, were separately sequenced using paired‐end reads (2 × 250 bp) on the Illumina MiSeq 2500 platform by Shanghai Biozeron Co., Ltd.

Standardized and previously described protocols were then used to process the raw sequence data [30, 31]. Briefly, in‐house Perl scripts were first used to demultiplex raw fastq files, and the following criteria were imposed to process barcode sequence information of each sample: (i) Reads (250 bp) were truncated at sites with an average quality score <20, determined over a 10 bp sliding window, and any segments <50 bp were discarded. (ii) Two nucleotide mismatches in primer matching and also reads that contained ambiguous characters were removed to ensure exact barcode matching. (iii) Only sequences that overlapped for >10 bp were assembled with reference to their overlapping sections, and reads that could not be assembled were discarded. The filtering and assembly of raw sequences were carried out using QIIME2 [39]. After quality filtering and chimera removal, a total of 2,991,546 clear reads of 16S rRNA V3–V4 amplicon sequences and 2,507,157 clear reads of ITS amplicon sequences were generated to survey the bacterial and fungal communities, respectively. Bacterial operational taxonomic units (OTUs) were taxonomically assigned using the RDP Classifier, referencing against the SILVA database [40] with a confidence threshold of 97%, whereas fungal OTUs were analyzed with the UCLUST algorithm and matched against the UNITE database [41] with a confidence threshold of 80%. Rarefaction curves indicated near‐saturation of community coverage (Supporting information: Figure S8). All samples were resampled to the same sequencing depth before microbial analysis.

### Fungal and bacterial community analysis

All statistical analyses were conducted in R version 3.6.2 (http://www.r-project.org/).

#### α‐diversity analysis

The “vegan” package was used to calculate three indices of alpha diversity: Pielou evenness, richness (observed number of OTUs), and the Shannon diversity index. A two‐way analysis of variance was applied to test whether insect species or sex had a significant impact on microbial Shannon diversity index. A nonparametric statistical test (*Kruskal–Wallis* test) was used to determine whether α‐diversity differed among host species groups. To test the correlations between bacterial and fungi α‐diversity indices within the fly or beetle, Pearson *r* and simple linear regression were used.

#### β‐diversity analysis

Bacterial and fungal community analyses were also conducted using “vegan,” and the results were then visualized using “ggplot2” [42]. Nonmetric multidimensional scaling (NMDS) analyses with Bray–Curtis distances were used to evaluate the dissimilarities in community composition between samples and assess their β‐diversity. To determine differences in community composition of bacterial and fungal microbiomes among samples, a permutational multivariate analysis of variance (PERMANOVA) was carried out with 9999 permutations using the *adonis* function [43].

#### Community composition analysis

The gut microbiome of the two insects was characterized based on relative abundances analyzed at multiple taxonomic levels. *Mann–Whitney U* tests were applied to detect significant differences in reading proportions of the bacterial 16S rRNA or fungal ITS genes at the genus level. Genera were considered differentially abundant when *p* < 0.05.

#### Co‐occurrence networks

To characterize significant relationships between the relative abundances of OTUs in bacterial and fungal communities of each sample, bacterial and fungal association networks were constructed using the “SpiecEasi” package and plotted using “ggClusterNet.” All strong correlations with *ρ* > |0.6| and *p* < 0.05 were considered statistically significant. The “vegan” and “igraph” packages were used to evaluate various network parameters including the clustering coefficient, average degree, and average path length, the network diameter, and the degree of centralization [44]. We also compared our resultant networks to a randomized version generated with the “igraph” package to identify any nonrandom patterns. Network complexity is reflected in the parameter “average degree,” where higher average degree values represent greater network complexity [45].

#### Microbial source‐tracking analysis

To determine the potential origins of fungi and bacteria found in the gut contents of the two insect species, we applied the Fast expectation‐maximization microbial source tracking (FEAST) method (https://github.com/cozygene/FEAST) using the “FEAST” package in R with default parameters [46]. FEAST is an expectation‐maximization‐based method that estimates the proportion of a microbial community that is the contribution of a potential source environment. The analysis enables a highly efficient estimation of the contribution of one habitat as a source to another as a sink.

#### Null model analysis

Null model analysis was carried out to quantify the relative contribution of ecological processes such as drift, selection, and dispersal in microbial assembly [47]. A fungal phylogenetic tree was constructed using Ghost tree (https://github.com/JTFouquier/q2-ghost-tree) [48], whereas a bacterial phylogenetic tree was constructed using FastTree2 [49]. Then, the beta Nearest Taxon Index (βNTI) was calculated by comparing the observed β‐mean nearest taxon distance (βMNTD) to the mean of a null distribution of βMNTD (999 randomizations). Standard deviations were normalized using the “picante” package in R. Large absolute values (|βNTI|≥ 2) represent deterministic processes having a dominant role in shaping microbial communities, while smaller absolute values (|βNTI| < 2) point to a stronger influence from stochastic processes instead. We then incorporated βNTI with Bray‐Curtis‐based Raup–Crick indices (RCI) to quantify the ecological processes, estimating the relative strength of homogeneous selection (βNTI < –2), variable selection (βNTI > 2), homogeneous dispersal (RCI < 0.95 and |βNTI| < 2), dispersal limitation (RCI > 0.95 and |βNTI| < 2), and drift (|RCI| < 0.95 and |βNTI| < 2) in driving the composition of the microbiota.

#### Functional prediction of the microbiome

Tax4fun [50] was used to predict the potential functions of gut microbial communities detected in each of the insect species. *Mann–Whitney* tests were used to investigate differences in pathways between the two insect species with reference to the Kyoto Encyclopedia of Genes and Genomes (KEGG). We used the FUNGuild program [51] to assign ecological functions to each fungal OTU. The OTU table was parsed against the FunGuild database to assign putative life strategies to taxonomically defined OTUs.

## AUTHOR CONTRIBUTIONS

Yu‐Xi Zhu and Yu‐Zhou Du designed the study. Yu‐Xi Zhu, Run Yang, Ming‐Hui Gong, Yuan Shen, Jue‐Ye Xu, and Xin‐Yu Wang conducted experiments. Tao Wen and Yu‐Xi Zhu analyzed the data. Yu‐Xi Zhu, Dian‐Shu Zhao, and Yu‐Zhou Du wrote the paper. All authors have revised, commented on, and approved of the final manuscript.

## CONFLICT OF INTEREST

The authors declare no conflict of interest.

## Supporting information

Supplementary information.

Supplementary information.

## Data Availability

The raw sequences of bacterial 16 S rRNA and fungal ITS genes have been deposited in the National Center for Biotechnology Information (NCBI) Sequence Read Archive (SRA) with the accession number PRJNA822659. R code used for data processing, graph creation, and statistical analyses related to this paper are available on GitHub (https://github.com/taowenmicro/zhu.et.al.2022.07).
