# Supplementary material for: Gut microbiota composition in the sympatric and diet‐sharing Drosophila simulans and Dicranocephalus wallichii bowringi shaped largely by community assembly processes rather than regional species pool
Source: Imeta. 2022 Oct 13;1(4):e57. doi: 10.1002/imt2.57 (PMC10989964; doi:10.1002/imt2.57)
Supplement: Supplementary file 1 — Supplementary information. [file IMT2-1-e57-s002.docx]

**Supporting Information for**

**Gut microbiota composition in the sympatric and diet-sharing *Drosophila simulans* and *Dicranocephalus wallichii bowringi* shaped largely by community assembly processes rather than regional species pool**

Yu-Xi Zhu^1^, Run Yang^1^, Xin-Yu Wang^1^, Tao Wen^2^, Ming-Hui Gong^3^, Yuan Shen^3^, Jue-Ye Xu^3^, Dian-Shu Zhao^4^, Yu-Zhou Du^1^*

^1^College of Plant Protection, Yangzhou University, Yangzhou, China

^2^The Key Laboratory of Plant Immunity, Jiangsu Provincial Key Lab for Organic Solid Waste Utilization, Jiangsu Collaborative Innovation Center for Solid Organic Wastes, Educational Ministry Engineering Center of Resource-saving fertilizers, Nanjing Agricultural University, Nanjing, China

^3^Bureau of Agriculture and Rural Affairs of Binhu District of Wuxi, Wuxi, China

^4^Entomology and Nematology Department, University of Florida, Gainesville, Florida, United States

***Correspondence**

Yu-Zhou Du, College of Plant Protection, Yangzhou University, 88 Daxue South Road, Hanjiang District, Yangzhou City, Jiangsu Province, 225009, China.

E-mail: [yzdu@yzu.edu.cn](mailto:yzdu@yzu.edu.cn)

**This file includes: Figure S1 to S8**


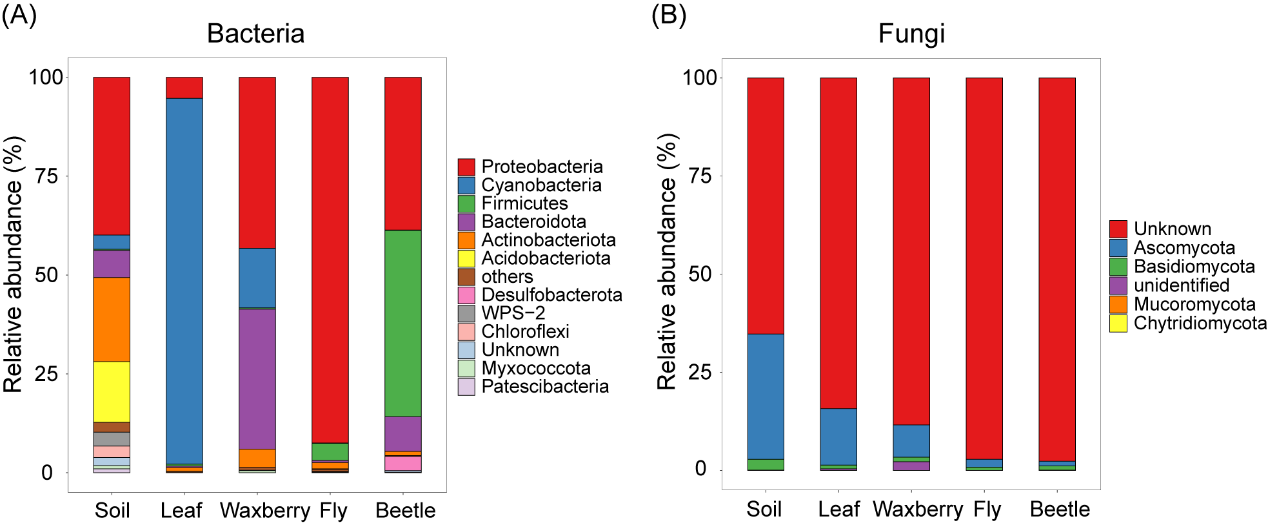


**Figure S1.** Variation in microbial composition among the fly *D. simulans*, the beetle *D. wallichii bowringi*, waxberry, leaves and soil. Relative abundances of bacterial (A) and fungal (B) communities at the phylum level in different groups.

**
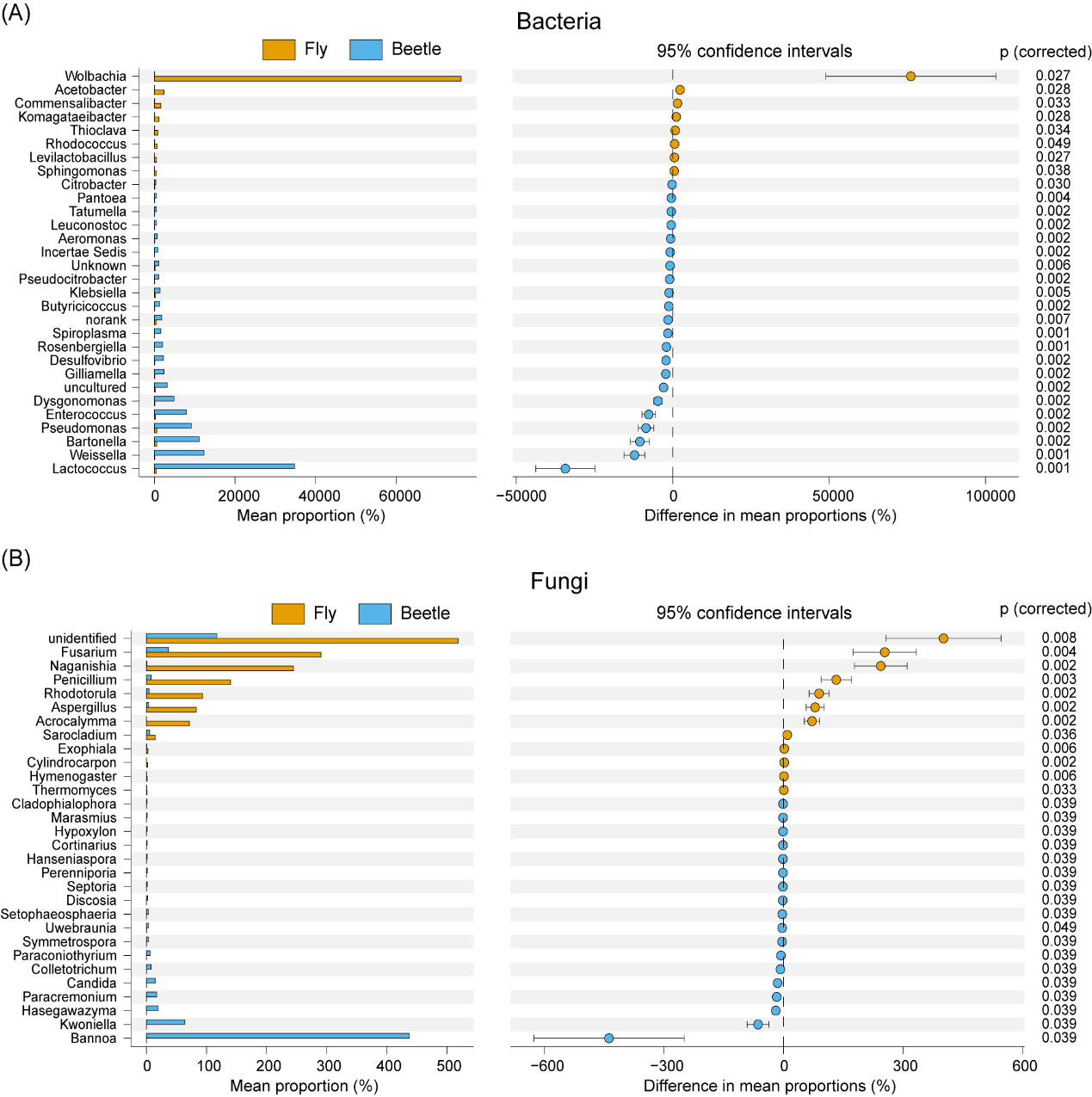
**

**Figure S2.** Read percentages of bacterial (A) and fungal (B) genera in the fly gut and beetle gut. Significant differences were determined using Mann–Whitney U tests and corrected values of the significance level *p* are shown on plot figures. Error bars represent 95% confidence intervals.

**
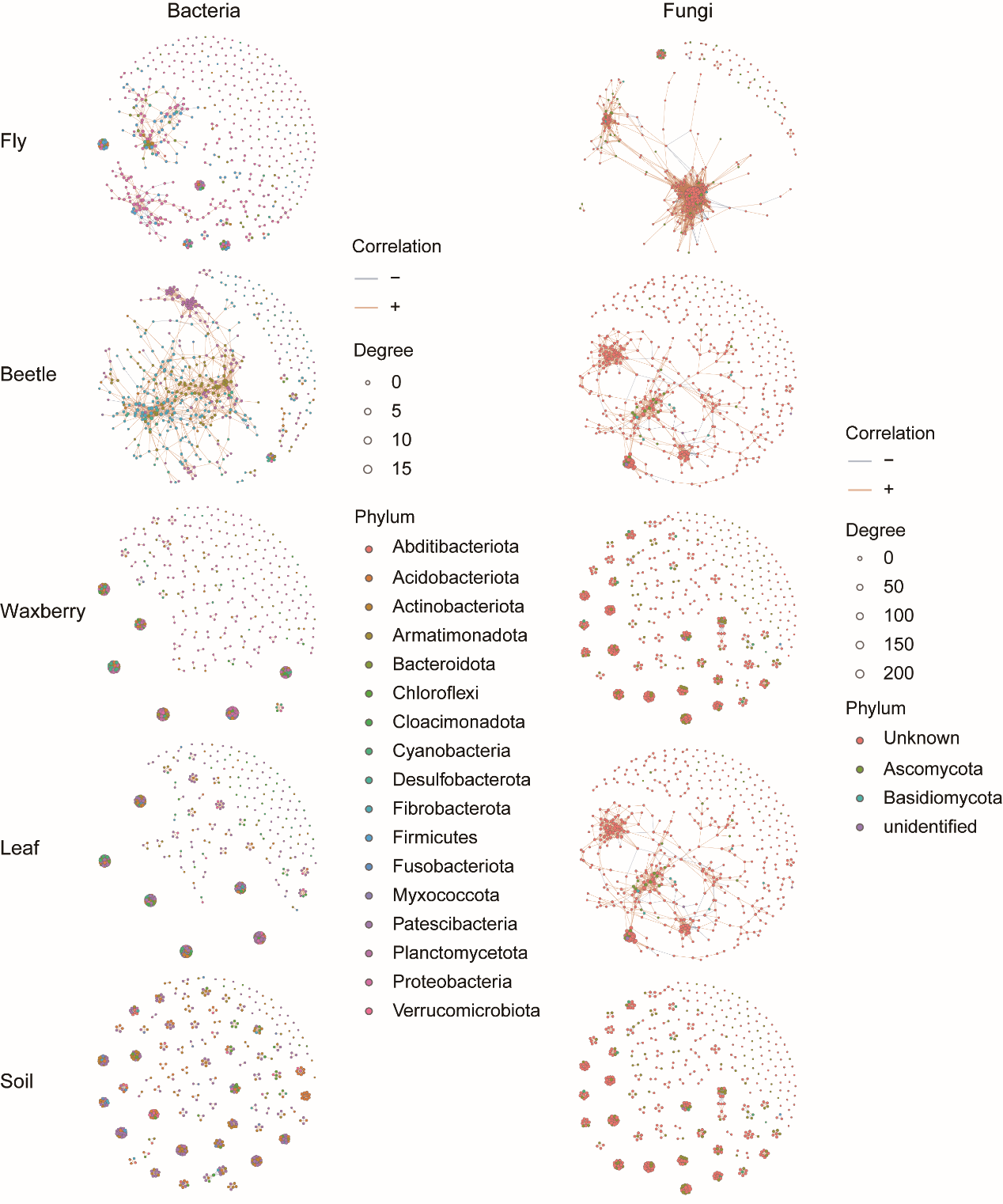
**

**Figure S3.** Co-occurrence networks of the microbial community in the fly *D. simulans*, the beetle *D. wallichii bowringi*, waxberry, leaves, and soil. Left and right panels indicating bacterial and fungal networks in different groups, respectively. Edges represent significant Spearman correlations (*ρ* > |0.6|, *p* < 0.05). Blue and red lines represent significant negative and positive correlations, respectively. The sizes of the points indicate the relative abundances of OTUs in each microbial community.

**Figure S4.** The relation between bacteria and fungi alpha diversity indices in the fly (A) and the beetle (B). Correlations are based on linear Pearson collection coefficients (*r*), and the significance level (*p*) is shown on plot figures.


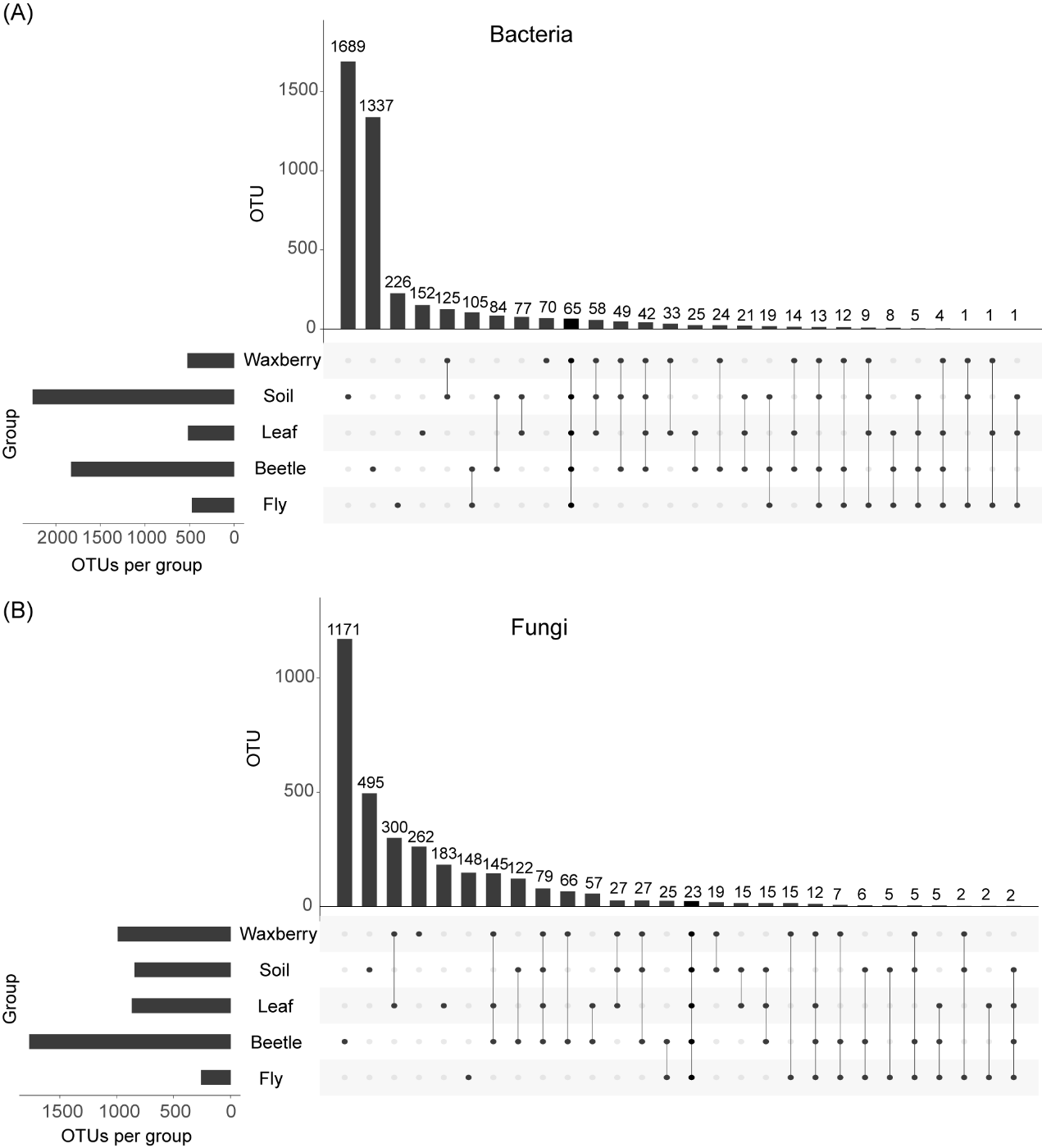


**Figure S5.** Distribution of shared bacterial (A) and fungal (B**)** OTUs among the fly *D. simulans*, the beetle *D. wallichii bowringi*, waxberry, leaves and soil.


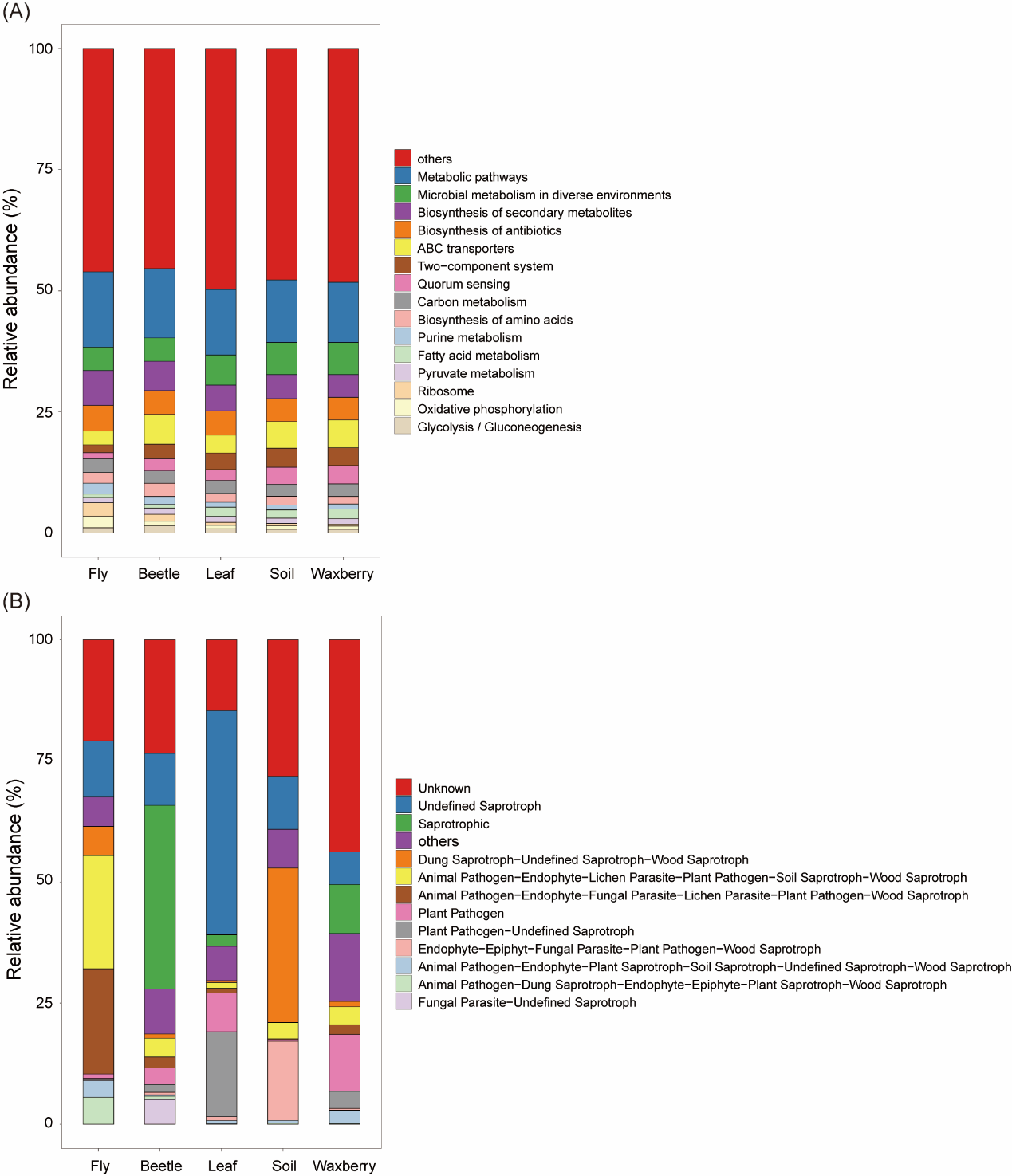


**Figure S6.** Functional prediction of the bacterial (A) and fungal (B) genes in different sample types.


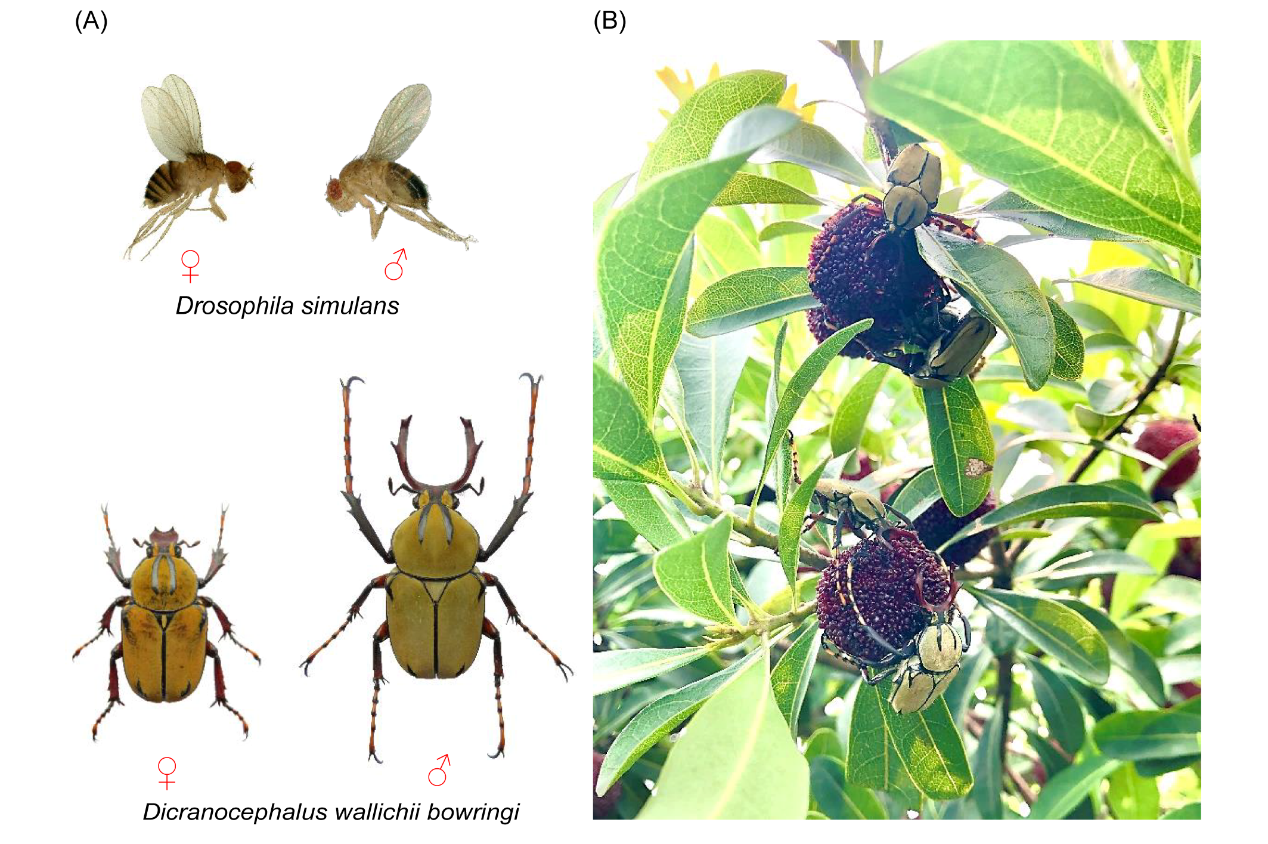


**Figure S7.** Adult female and male of *Drosophila simulans* and *Dicranocephalus wallichii bowringi* (A) and their habitat (B). The photos were taken with a Leica camera (DVN6a) or iPhone.

**
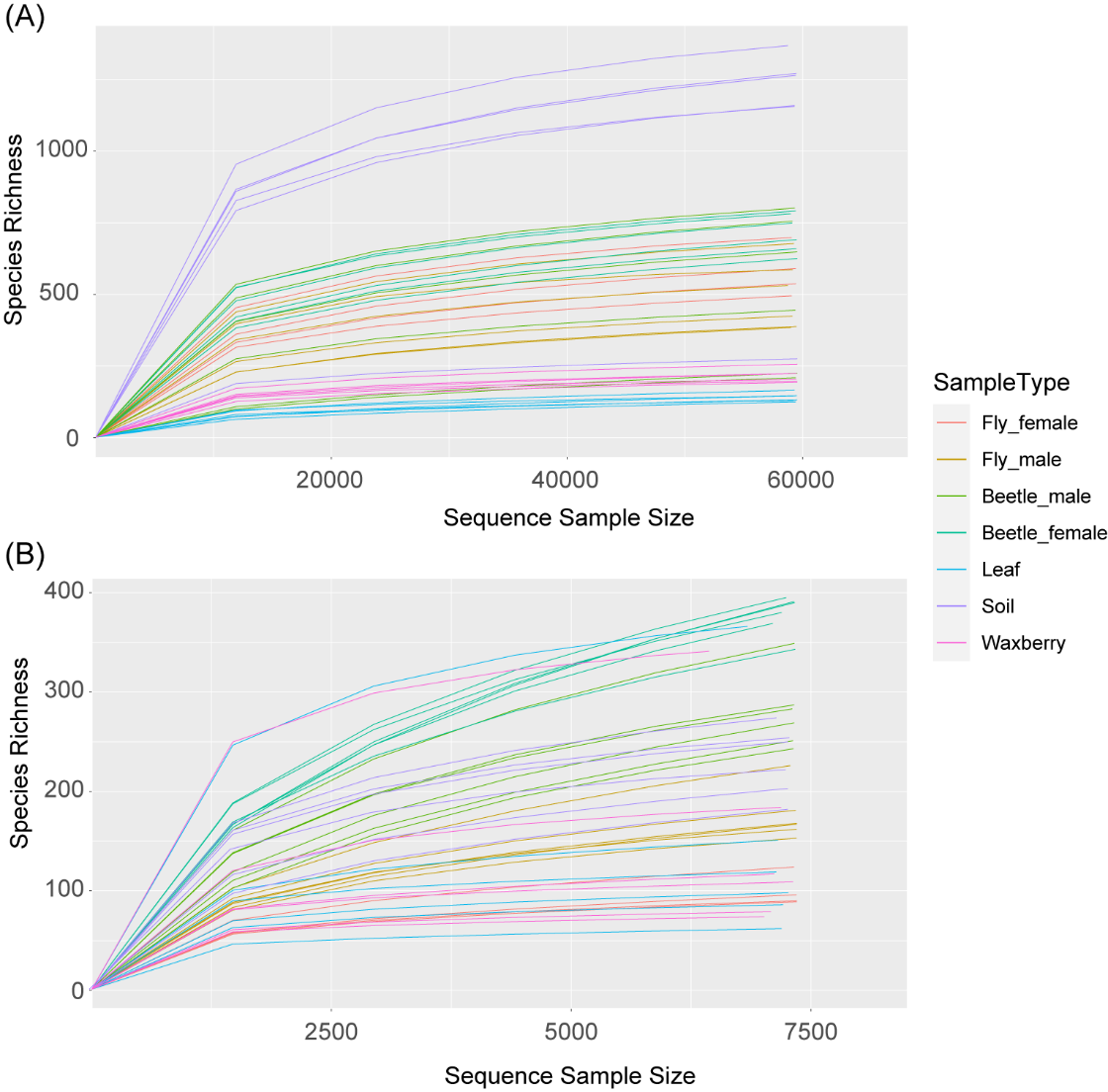
**

**Figure S8.** Rarefaction curves for bacterial (A) and fungal (B) communities in *D. simulans*, *D. wallichii bowringi*, waxberry, leaves, and soil.
